# A Case of Contact Allergic Dermatitis to Topical Minoxidil

**DOI:** 10.7759/cureus.12510

**Published:** 2021-01-05

**Authors:** Hessah BinJadeed, Almuntsrbellah M Almudimeegh, Shadn A Alomran, Abdullah H Alshathry

**Affiliations:** 1 Dermatology, King Khalid University Hospital, Riyadh, SAU; 2 College of Medicine, King Saud University, Riyadh, SAU

**Keywords:** minoxidil, contact allergic dermatitis, androgenic alopecia, female pattern hair loss, angioedema

## Abstract

Coincidental findings of hypertrichosis in patients on minoxidil led to the development of a topical minoxidil formulation which has been approved by the Food and Drug Administration for the treatment of female pattern hair loss, the most common cause for hair loss in women. The most common side effect of topical minoxidil is irritant contact dermatitis with the typical symptoms of itching and scaling. Most commonly, these symptoms are a result of an allergic reaction to propylene glycol, or less commonly, to minoxidil itself. We present a case of a 27-year-old woman who developed facial swelling following allergic contact dermatitis to minoxidil 5% foam.

## Introduction

Female pattern hair loss (FPHL) or androgenetic alopecia is the most common cause of hair loss in women and one of the most frequent chronic conditions encountered by dermatologists worldwide [1]. FPHL is nonscarring alopecia characterized by hair follicle miniaturization, a progressive transformation of pigmented terminal hair into nonpigmented villous hair [2-5]. The cause for miniaturization remains uncertain but is postulated to be a combination of genetic predisposition, androgen effect, and other not well-understood factors [3-5]. Minoxidil was first introduced in the 1970s as an oral hypertensive medication and a potent vasodilator [6]. Coincidental findings of hypertrichosis in patients on minoxidil led to the development of a topical minoxidil formulation, which has been approved by the Food and Drug Administration (FDA) for the treatment of FPHL in 1992 [7]. It is available over the counter with a good safety profile [7]. The most common side effect of topical minoxidil is irritant contact dermatitis with the typical symptoms of itching and scaling [8]. Most commonly, these symptoms are a result of an allergic reaction to propylene glycol, or less commonly, to minoxidil itself [8]. We present a case of a 27-year-old woman who developed allergic contact dermatitis to minoxidil 5% foam. In addition to our case, a few other instances of allergic reactions to minoxidil itself have been reported in the literature [8-10].

## Case presentation

**Figure 1:**
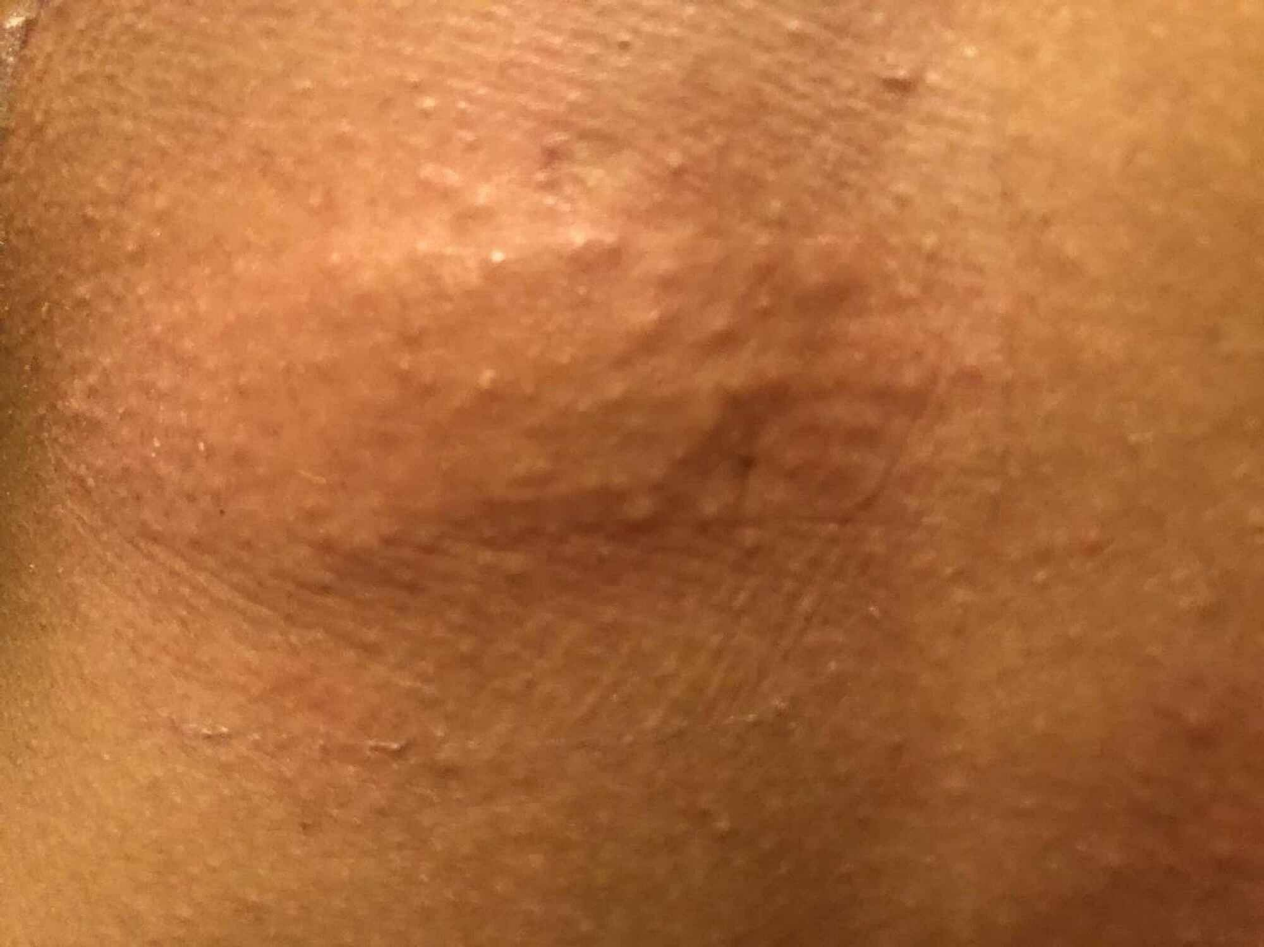
Positive patch test to minoxidil 5%.

This is a case of a 27-year-old woman, not known to have any medical illnesses, who presented to our dermatology clinic with a painful swelling over the face for two days. Upon further inquiry, she used topical minoxidil 5% foam for FPHL applied once daily for four days prior to her presentation. After applying the foam, she reported itchiness over the scalp, and therefore, she stopped using it. Regardless, the itchiness continued, and she started to develop gradual swelling over the face. She presented to the emergency department one day prior to her appointment in the dermatology clinic and was given intramuscular diphenhydramine 50mg and oral prednisone 30mg. The patient had previously been prescribed minoxidil 5% spray for FPHL, and she reported itchiness over the scalp. She was then advised to substitute it with minoxidil 5% foam (propylene glycol free) after which, she presented with her current complaint. A patch test was done with a minoxidil solution containing 5% minoxidil in alcohol, and the results were interpreted by an immunologist, which showed positive sensitization (Figure 1). A patch test to alcohol and propylene glycol showed no reaction. Consequently, she was advised to avoid topical minoxidil solution or foam as a treatment for her FPHL.

## Discussion

FPHL is a nonscarring type of alopecia in which there is progressive hair thinning at the scalp vertex with a spared frontal hairline. This occurs secondary to changes in the hair cycle with simultaneous shortening and lengthening of the anagen and telogen phases, respectively. This results in hair follicle miniaturization, a progressive transformation of pigmented terminal hair into nonpigmented villous hair [2-5,11,12]. Oral minoxidil is a piperidino-pyrimidine derivative, a vasodilator that is used for the treatment of hypertension. When applied topically, it has shown a direct stimulatory effect on dermal papillae and follicular hair matrix cells that is effective in arresting the process of miniaturization and producing some degree of regrowth [13]. Topical minoxidil solution is currently available over the counter and has a considerably good safety profile [8]. The most common side effects of topical minoxidil include exacerbation of seborrheic dermatitis, irritant contact dermatitis, or allergic contact dermatitis [8]. A patch test can be provided to patients with allergic contact dermatitis to determine the causative allergen [8]. The first case of allergic contact dermatitis to 1% minoxidil solution was reported by Weiss et al. in 1984 in a patient treated for alopecia areata [14]. Since then, other cases of contact dermatitis as an adverse event to topical minoxidil have been described in the literature. These instances have been commonly reported to be due to minoxidil solution vehicles such as propylene glycol and less frequently to minoxidil itself, like in our case [8-10]. Rarely, oral minoxidil has been associated with rashes, bullous eruptions, and Stevens-Johnson syndrome [15]. If patients are found to be allergic to propylene glycol, compounded formulations with alternative solvents can be used. However, patients who are found to be allergic to minoxidil itself are not candidates for using minoxidil to treat their alopecia [8]. Oral minoxidil has a vasodilatory effect, which may lead to angioedema as a dose-dependent side effect [16]. Frontal edema following application of topical minoxidil has been reported; however, it was applied following hundreds of mesotherapy injections, which most likely increased the absorption of topical minoxidil [17]. In our case, the patient was previously sensitized to topical minoxidil. Two days after the application of topical minoxidil foam, she developed gradual facial edema, which mimicked angioedema; allergic contact dermatitis was proven by patch testing. An instance of the so-called pseudoangioedema, An allergic contact dermatitis mimicking angioedema, was also reported in the literature [18].

## Conclusions

Allergic contact dermatitis to minoxidil itself is increasingly reported in the literature. Therefore, patch testing should be considered if the patient reports itchiness or erythema following the application of topical minoxidil. Patients who experience an allergic reaction to topical minoxidil are commonly advised to switch to minoxidil foam as it is propylene glycol free. However, patients who are found to be allergic to minoxidil itself are not candidates for using topical minoxidil to treat their alopecia. Other treatments can be used off-label, such as 5α-reductase inhibitors, androgen receptor antagonists, prostaglandin analogs and antagonists, laser therapy, and platelet-rich plasma injections.
